# In Situ Growth of a High-Performance All-Solid-State Electrode for Flexible Supercapacitors Based on a PANI/CNT/EVA Composite

**DOI:** 10.3390/polym11010178

**Published:** 2019-01-21

**Authors:** Xipeng Guan, Debin Kong, Qin Huang, Lin Cao, Peng Zhang, Huaijun Lin, Zhidan Lin, Hong Yuan

**Affiliations:** 1 School of Mechanics and Construction Engineering, Jinan University, Guangzhou 510632, China; guanxipeng@jnu.edu.cn; 2 Institute of Advances Wear & Corrosion Resistant and Functional Materials, Jinan University, Guangzhou 510632, China; 13580457530@163.com (D.K.); hq2502@126.com (Q.H.); linc19993@163.com (L.C.); tzhangpeng@jnu.edu.cn (P.Z.); hjlin@jnu.edu.cn (H.L.)

**Keywords:** PANI, CNT, Flexible supercapacitor electrode, Polymer conductive film

## Abstract

For the development of light, flexible, and wearable electronic devices, it is crucial to develop energy storage components combining high capacity and flexibility. Herein, an all-solid-state supercapacitor is prepared through an in situ growth method. The electrode contains polyaniline deposited on a carbon nanotube and a poly (ethylene-co-vinyl acetate) film. The hybrid electrode exhibits excellent mechanical and electrochemical performance. The optimized few-layer polyaniline wrapping layer provides a conductive network that effectively enhances the cycling stability, as 66.4% of the starting capacitance is maintained after 3000 charge/discharge cycles. Furthermore, the polyaniline (PANI)-50 displays the highest areal energy density of 83.6 mWh·cm^−2^, with an areal power density of 1000 mW·cm^−2^, and a high areal capacity of 620 mF cm^−2^. The assembled device delivers a high areal capacity (192.3 mF·cm^−2^) at the current density of 0.1 mA·cm^−2^, a high areal energy (26.7 mWh·cm^−2^) at the power density of 100 mW·cm^−2^, and shows no significant decrease in the performance with a bending angle of 180°. This unique flexible supercapacitor thus exhibits great potential for wearable electronics.

## 1. Introduction

With the explosive development of light, flexible, and wearable electronic devices, supercapacitors have become a new type of energy storage device, attracting a lot of attention in academia [1,2,3,4,5]. It is believed that these electronic devices will bring important changes to human lifestyles in the future [6,7,8,9]. In order to support the development of these electronic devices, energy storage devices should have good electrochemical performance and outstanding flexibility. Supercapacitors are superior to other power storage devices, mainly due to their high power density, fast charge and discharge rates, flexibility, lightness, low cost, and environmental friendliness [10,11]. In the past decade, supercapacitors have achieved great advances in high energy density features and high power density features from the development of new energy materials [12].

However, the current commercially available supercapacitor and battery systems are still rigid and cumbersome. It is difficult to incorporate them into complex devices, which usually leads to bulkiness, low energy density, and poor wearability; thus, this becomes a bottleneck for the further development of wearable electronics [13]. The composition and structure of the electrode material directly determine the energy storage performance of a supercapacitor. Traditional flexible supercapacitors are made by mixing active materials, conductive agents, and adhesives, and then coating them on foam nickel. The disordered dispersion of the conductive agents and adhesives in the active materials reduces the effective specific surface area, ion transport capacity, material lifetime, capacitance, and energy–density ratio. The challenges of supercapacitors for future wearable applications can be divided into six aspects: Energy density and power density [14,15], long-term durability [12,16,17], wearability [18,19], safety [20,21,22,23,24,25], washability [23,26], and scalability [17,24,27,28].

Recently, the integration of functional materials—including carbon nanotubes, metal oxides, and conductive polymers—into various flexible substrates such as textiles, papers, and plastics, was achieved to fabricate flexible supercapacitor electrodes [29,30,31,32,33]. Among these substrates, a film that is composed of composite conductive polymer exhibits a set of properties that is different from that of paper used as a conventional flexible substrate, due to the natural features of ethylene-vinyl acetate copolymer (EVA). EVA is a new environmentally friendly plastic foam material, which has good buffering, shock resistance, heat insulation, moisture resistance and chemical corrosion resistance, and is nontoxic and nonabsorbent.

Conductive polymers, such as polyaniline (PANI), polythiophene, and polypyrrole (PPy), have been used for fabricating flexible electrodes [34,35,36]. In particular, PANI has received special attention due to its excellent specific capacitance, cost effectiveness, ease of use, and favorable stability performance. However, PANI experiences large volumetric swelling and shrinkage during the charge–discharge process because of ion doping and de-doping, which results in structural breakdown and thus fast capacitance decay of the conducting polymers [37]. Up to now, most reports of PANI electrodes report no more than 80% after 1000 cycles [38,39,40,41,42].

Studies have shown that low-dimensional nanomaterials with nanostructured PANI, including nanorods, nanotubes, and nanospheres, have yielded superior capacitive performance in comparison with pure PANI and similar carbonaceous counterparts [30,35,43]. However, the rigidity of the PANI molecular chain and the interaction between the molecular chains make it extremely low in solubility and almost insoluble in any organic solvent. This can lead to poor film-forming properties and processing properties of doped polyaniline, which seriously hinders its large-scale popularization and application in various fields.

In order to solve the problem mentioned above, we report a process for the design and fabrication of PANI/CNT(conductive phase)/EVA polymer conductive film to obtain a modified, flexible, bendable, and good conductive surface of the supercapacitor electrode. The research mainly focuses on three aspects: (1) Adding the conductive phase (CNT) to enhance the EVA film material’s ion transmission capacity; (2) examining how the nanostructure design of the PANI can increase the effective specific surface area and specific capacitance; and (3) choosing the facile electrodeposition method to increase the binding force between the active material and the collector. Herein, the morphologies, microstructures, and electrochemical performances of the resulting PANI/CNT/EVA products are investigated. The prepared flexible PANI/CNT/EVA composite materials were used to fabricate symmetric, flexible, solid-state symmetric supercapacitors with aqueous electrolytes. This work is believed to offer new scope for diversified applications of fabricated flexible electrodes in wearable energy storage devices.

## 2. Materials and Methods

### 2.1. Materials

All the chemicals used in this study were of analytical reagent grade. H_2_SO_4_ was obtained from Guangzhou Chemical Reagent Factory (Guangzhou, China). Poly(vinyl alcohol) (PVA) and poly(ethylene–co–vinyl acetate) (EVA) were purchased from Shanghai Macklin Biochemical Co., Ltd. (Shanghai, China). Sodium dodecylbenzene sulfonate (SDBS), tetrahydrofuran (THF), and aniline were provided by the Tianjin Damao Chemical Reagent Factory (Tianjin, China). CNT was purchased from Korea Kumho Corporation (Seoul, Korea). Meanwhile, 331 carbon cloth was obtained from Shanghai Fanyue Electronic Technology Co., Ltd. (Shanghai, China). 

### 2.2. Preparation of CNT/EVA and CNT/EVA Cotton

To prepare Solution A, 0.10 g of SDBS was dissolved in 100 mL tetrahydrofuran (THF), after which 0.15 g of CNT was slowly added to the solution and this was followed by ultrasonication for 0.5 h (ultrasonic power of 100 W, ultrasonic frequency of 80 kHz). To prepare Solution B, 0.5 g EVA (VA value 32%) was dissolved in 50 mL THF. Solutions A and B were mixed and then ultrasonically dispersed for 30 min. The mixed solution was poured into a petri dish, and the CNT/EVA film was fabricated after volatilization in the fume cupboard for 2 h. The cotton was immersed in CNT/SDBS/EVA/THF mixed solution, the CNT/EVA cotton conductive material was realized by the surface modification of cotton cores with the CNT/SDBS/EVA/THF mixed solution.

### 2.3. Preparation of CNT Paper

The paper was immersed in the CNT/SDBS suspension solution, and CNT successfully coated the fiber surface to obtain CNT paper.

### 2.4. Preparation of CNT/EVA/PANI Composite Electrodes

PANI was grown on the CNT/EVA film substrate by a facile electrodeposition process. A piece of CNT/EVA film (ca. 1 × 1 cm) was cut and carefully cleaned in ultrapure water several times and completely dried at 60 °C before use. The electrodeposition process was conducted in a three-electrode system with the CNT/EVA film substrate as the working electrode, a saturated calomel electrode as the reference electrode, and a platinum electrode as the counter electrode. The electrochemical deposition of PANI was carried out under cyclic voltammetry of 1 V (−0.2 to 0.8 V) in an aqueous solution containing 0.1 M aniline monomer and 1 M H_2_SO_4_. After electrodeposition, the sample was rinsed with ultrapure water several times and completely dried at 60 °C. The prepared CNT/EVA/PANI film electrodes with PANI deposition times of 5, 10, 20, 30, and 50 were denoted PANI–5, PANI–10, PANI–20, PANI–30, and PANI–50, respectively.

### 2.5. Solid-State Supercapacitors were Assembled with a PVA/H_2_SO_4_ Gel Electrolyte

The gel electrolyte was prepared by stirring a mixture of 5 g of PVA, 5 g of H_2_SO_4_, and 50 mL of deionized water at 85 °C. for 2 h. The electrodes and separator were immersed into the gel electrolyte for several minutes. A solid-state symmetric film supercapacitor was assembled by sandwiching the diaphragm paper into two identical PANI/CNT/EVA composite electrodes.

### 2.6. Characterization Techniques

Morphological observations were carried on using a field emission scanning electron microscope (FE-SEM, Zeiss, Oberkochen, Germany) at an operating voltage of 10 kV.

Galvanostatic charge-discharge (GCD), cyclic voltammetry (CV), and electrochemical impedance spectroscopy (EIS) were performed on the flexible electrode using a Princeton (PARSTAT 4000) electrochemical station. The capacitances (C in F·cm^−2^) of each device at different current densities were calculated from the discharge curves obtained from GCD tests using the formula

(1) C=2IΔt/SΔV

where *I* is the applied discharge current (A), Δ*t* is the discharge time (s), and Δ*U* (V) is the discharge voltage after the IR drop is removed. *S* (cm^2^) is the volume of the active materials of all electrodes. Here, the surface areas of the active materials are 1 cm^2^.

The volumetric energy density and power density can provide more reliable performance metrics for porous nanomaterial-based thin-film devices compared to gravimetric capacitance. As a result, the volumetric energy density (Wh·cm^−2^) of each device was calculated using [44]

(2) $$E=0.5C\Delta U^{2}/3.6$$

The volumetric power density (W·cm^−2^) of the device was calculated from

(3) P=3600E/Δt

We investigated the electrochemical properties of all electrodes on a three-electrode system using a Pt plate as the counter electrode and a saturated calomel reference electrode (SCE) in 1 M H_2_SO_4_ aqueous electrolyte solution.

## 3. Results and Discussion

**Figure 1:**
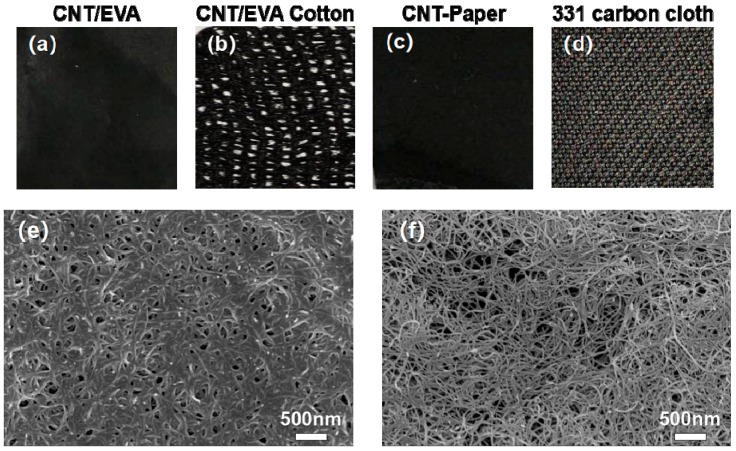
Digital photographs of four different electrodes: (**a**) conductive phase (CNT)/EVA, (**b**) CNT/EVA Cotton, (**c**) CNT paper, (**d**) 331 carbon cloth. SEM images of (**e**) CNT/EVA and (**f**) CNT paper.

Figure 1a–d show digital photographs of three different experimental electrode preparations (CNT/EVA film, CNT/EVA cotton, CNT paper) and one sample of commercial 331 carbon cloth. The morphologies of different textile electrodes were investigated by SEM. The CNT/EVA aqueous suspension was dropped into a petri dish and the CNT/EVA film was obtained after volatilization. Figure 1e shows the SEM of CNT/EVA film. In Figure 1e the carbon fibers display a smooth and defect free surface morphology, providing high electron conductivity and robust mechanical properties. Secondly, converting the medical gauze to a conductive material was realized by the surface modification of medical gauze cores with EVA/CNT to form CNT/EVA cotton. There are numerous micropores and mesopores in the CNT/EVA cotton, as shown in Figure S1. The conductive path of CNT/EVA cotton was incomplete. In previous experiments, the CNT aqueous suspension was first dropped onto the surface of paper until the CNT dispersion was saturated to obtain CNT paper. Figure 1f shows that the 3D network architecture of the CNT paper has a lot of holes, which is based on overlapped and entangled carbon fibers. The woven texture of the 331 carbon cloth can be observed in Figure S2. There are many parallel grooves and small bulging spots spreading on the surface of activated carbon fibers. The conductive path of 331 carbon cloth was complete, but this material was prone to brittle fracture.

**Figure 2:**
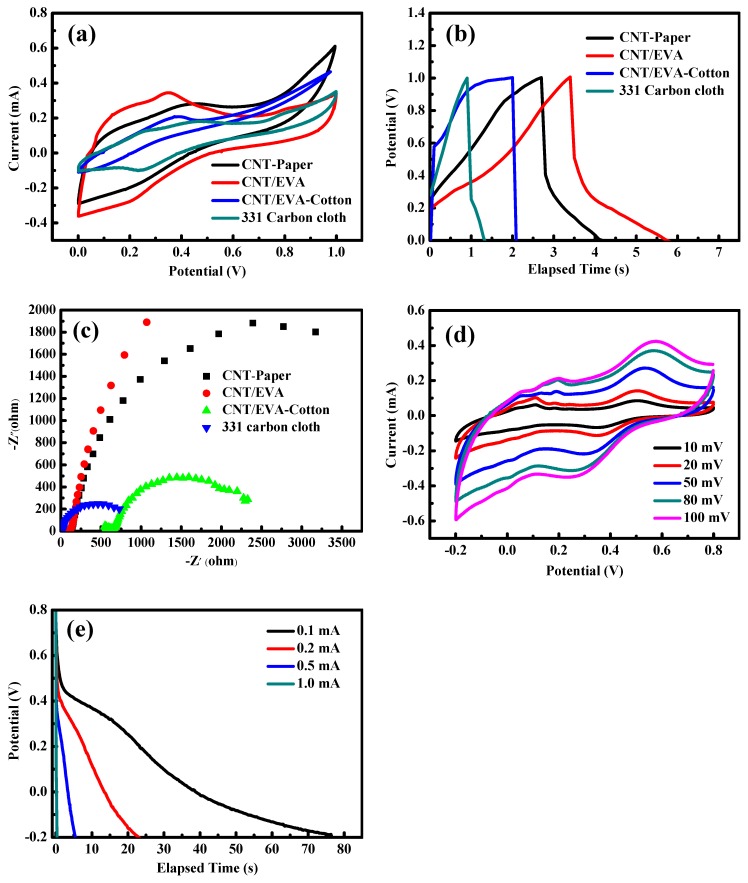
(**a**) Cyclic voltammetry (CV) curves (100 mV·s^−1^), (**b**) galvanostatic charge-discharge (GCD) curve (0.5 mA·cm^2^), and (**c**) electrochemical impedance spectroscopy (EIS) measurement for flexible electrodes made from different carbon materials. (**d**) CV curves and (**e**) discharge curve for the CNT/EVA electrode.

The cyclic voltammetry (CV) test curve can reflect the ion orientation and charge transfer at the electrode and electrolyte interface in the capacitor. The size of the integral area around the CV curve reflects the capacitance of the electrode material. The CV curves of the different carbon material flexible electrodes are shown in Figure 2a for the sweep speed of 100 mV·s ^−1^. Figure 2a shows that the CNT/EVA electrode exhibited the largest CV curve area when compared to the CNT/EV cotton, CNT paper, and 331 carbon cloth electrodes. The discharge curves of the CNT/EVA, CNT/EVA cotton, CNT paper, and 331 carbon cloth electrodes at the same current density of 0.5 mA·cm^2^ are shown in Figure 2b. Among these four electrodes, the CNT/EVA electrode exhibited the longest discharge time. When these electrodes were under 0.5 mA·cm^2^ given current density, the specific capacitance values were 3.1, 2.5, 0.1, and 0.7 mF·cm^2^, respectively. Furthermore, to gain insight into the carrier transport efficiency of the carbon material flexible electrodes, Figure 2c shows the Nyquist curves in the frequency ranged from 100 mHz to 100 kHz for the CNT/EVA, CNT/EVA cotton, CNT paper, and 331 carbon cloth electrodes. Their charge transfer resistances, obtained from the diameters of the semicircles at high frequency, were 74.4, 542.2, 111.2, and 9.16 Ω. CNT/EVA displays the smallest semicircles and the steepest vertical line, attesting to its low resistance and rapid capacitive response. The other samples have larger semicircles, and the slopes in the high frequency region are closer to the 45°line, revealing an increasing resistance due to diffusion–limited electrochemistry reactions. Test curves for different scan rates can show the response ability of the capacitor electrode to changing voltage. If the CV curve shape remains unchanged under a high speed, then the electrode has a good response performance to voltage and a good performance ratio. In order to verify the performance of the CNT/EVA electrode, we performed CV testing on the electrode at different scanning rates (Figure 2d). In the −0.2 to 0.8 V voltage range, the shape of the CV curve remains the same with increasing scanning speed. Calculations based on the discharge graph (Equation (1)) indicate that the CNT/EVA electrode areal capacitance is between 7.66 mF·cm^2^ and 0.8 mF·cm^2^ at current densities of 0.1 to 1 mA·cm^−2^ (Figure 2e). This value is much higher than for the CNT/EVA cotton, CNT paper, and 331 carbon cloth electrodes. 

**Figure 3:**
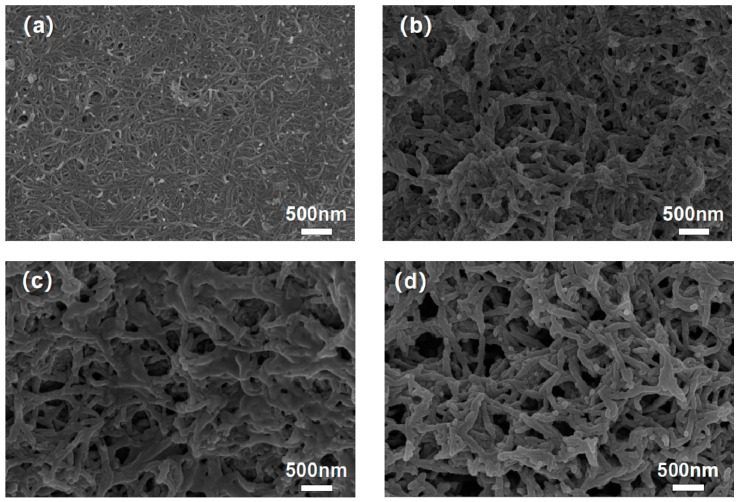

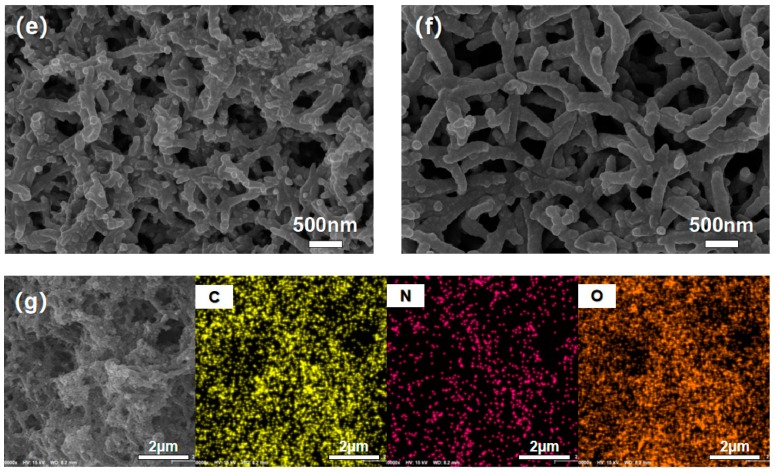
SEM images of (**a**) CNT/EVA, (**b**) PANI-5, (**c**) PANI-10, (**d**) PANI-20, (**e**) PANI-30, and (**f**) PANI-50 electrodes. (**g**) EDS analysis of PANI–5, C element, N element, and O element.

After drying the CNT/EVA film, an electrochemical polymerization process was used to deposit PANI on the CNT and EVA surface in the CNT/EVA film to obtain a PANI/CNT/EVA flexible electrode. The “dipping, drying, and electrochemical polymerization” process is similar to layer-by-layer assembly. Figure 3b–g shows the structure of the PANI/CNT composite in which the carbon fibers are uniformly covered with PANI particles. The PANI particles show a short, stick-like morphology. This was beneficial for increasing the specific surface area of the whole electrode. The PANI electrochemical deposition times were 5, 10, 20, 30, and 50, respectively. Figure 3b shows that a net-like structure was formed when the PANI deposition time was 5, and the average diameter size was 60 nm. When the PANI deposition time was increased to 10 (as shown in Figure 3c), some of the PANI nanorods connected, and the average diameter size was 70 nm. The surface generated a secondary growth when the PANI deposition time was increased to 20 (Figure 3d), and the average diameter size was 80 nm. When the PANI deposition time reached 30 (Figure 3e), a number of secondary growth points much larger than that of the former one occurred. At a deposition time of 50, the nanorod volume of the nanotubes was further dilated, which may have been caused by growth of the surface layer; the average diameter size was 120 nm. In addition, Figure 3g shows the chemical composition as investigated by energy dispersive spectrum (EDS) mapping, showing the distribution of carbon, nitrogen, and oxygen (C, N, and O) over the corresponding SEM picture. Since nitrogen followed the same distribution as C and O, it is believed that the PANI covers the whole CNT/EVA surface.

**Figure 4:**
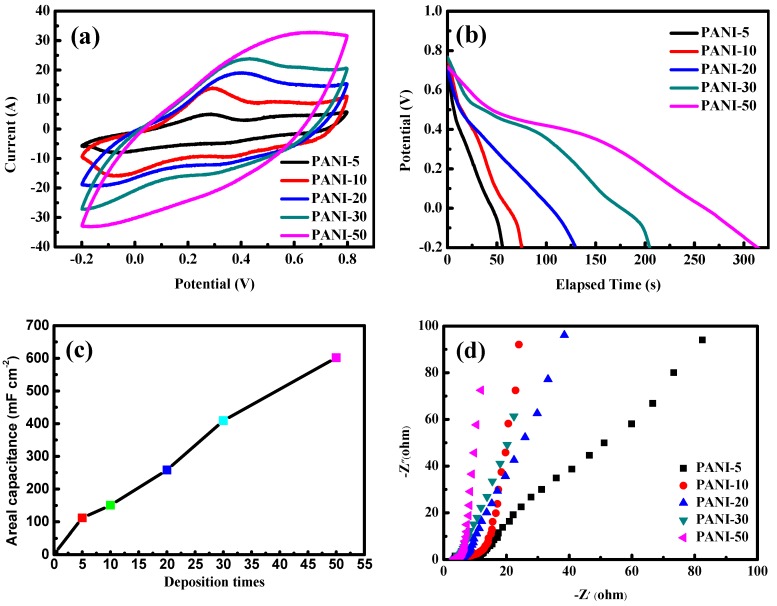

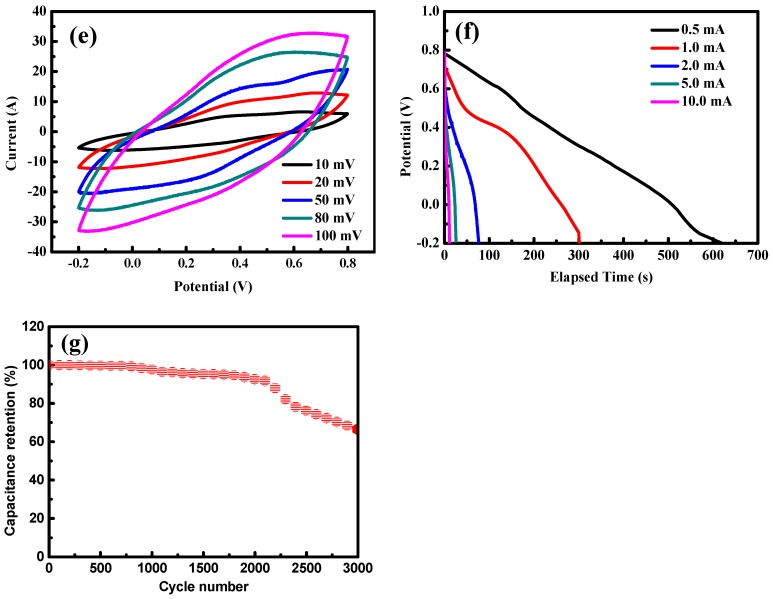
(**a**) CV (100 mV·s^–1^), (**b**) discharge curves (1 mA·cm^–2^), (**c**) areal capacitance and (**d**), EIS measurements of the PANI 5, 10, 20, 30, and 50 electrodes; (**e**) CV and (**f**) discharge curves of PANI–50 electrode at different scan rates and current densities; and (**g**) cycling stability of the PANI-50 electrode.

The flexible electrode exhibits outstanding performance in areal capacitance, which is a key parameter for applications in flexible and wearable electronics. One redox peak was observed in the CV curve of the conventional supercapacitor based on the PANI/CNT/EVA composite flexible electrode (Figure 4a), due to the faradaic reaction from PANI, corresponding to its emeraldine/pernigraniline structural conversions [45], as shown in Figure 4b. With an increase of the PANI deposition time, the electrode material needed a longer discharge time, which indicated that the electrode material had a larger capacity to store energy. The ratio of capacitance can be calculated from the discharge curve, as shown in Figure 4c. The specific capacitance values of PANI/CNT/EVA were 55.8, 75.2, 210.4, 289.0, and 310.4 mF·cm^2^ when the PANI deposition times were 5, 10, 20, 30, and 50, respectively. The specific capacitance of PANI/CNT/EVA was much larger than that of n CNT/EVA film (mF·cm^–2^), as shown in Figure 2e. This result suggests that the specific capacitance of PANI/CNT/EVA was remarkably improved due to its additional pseudocapacitance, which originated from PANI. Figure 4c shows that further increasing the PANI deposition time lead to a decrease in the growth rate of specific capacitance for PANI/CNT/EVA. This trend of the composite electrode may be because PANI volumetrically swells with increasing deposition time, and the impedance of the composite electrode is decreased (Figure 4d) due to the deposition of more PANI. The semicircle diameter of the high frequency zone is small, indicating that the internal resistance of the electrode material is very small after the modification of PANI deposition. The straight line is close to 90°in the low frequency area, showing that the electrode capacitance performance is better. The Nyquist plots in Figure 4d illustrate the impedance characteristics of the PANI/CNT/EVA electrodes in 1 M H_2_SO_4_ solution. The Rct values of the electrodes with different PANI deposition are all below 4Ω; these values can be employed to assess the charge transfer ability of the electrodes. A lower Rct corresponds to facile charge transfer kinetics within electrodes; therefore, the PANI–50 electrode has the highest charge transfer capacity. In order to verify the performance of the PANI-50 electrode, we performed CV testing on the electrode at different scanning rates (Figure 4e). In the −0.2 to 0.8 V voltage range, the shape of the CV curve remains the same with increasing scanning speed. The PANI-50 electrode CV curves maintained a good rectangular shape with a larger integral area at a high scan speed (100 mV/s). This shows that the electrode response performance to voltage variation was relatively good and had a good performance ratio. The discharge curve of the PANI-50 electrode at different current densities is shown in Figure 4f. When the electrodes were under 0.5, 1.0, 2.0, 5.0, and 10.0 mA·cm^−2^ given current density, the specific capacitance values were 620, 602, 306, 256, and 224 mF·cm^−2^, respectively. Figure 4g shows the excellent cycling performance of PANI-50 after 3000 cycles at a current density of 5 mA·cm^−2^. The adhesion of PANI to the surface of CNT/EVA seems to be extremely robust, as shown in Figure S3. After the cycling, no significant flaking off was observed. The PANI-50 sample retains up to 97.6% of its initial capacitance after the first 1000 cycles (Figure S4). After 3000 cycles, it can retain 66.4% of its initial capacitance. The PANI-50 exhibits excellent long-term stability. The high capacitance retention of PANI-50 over 3000 cycles is ascribed to the effective contact decrease between unstable PANI particles. The PANI/CNT/EVA composite electrode shows better cycling stability and capacitance retention.

**Figure 5:**
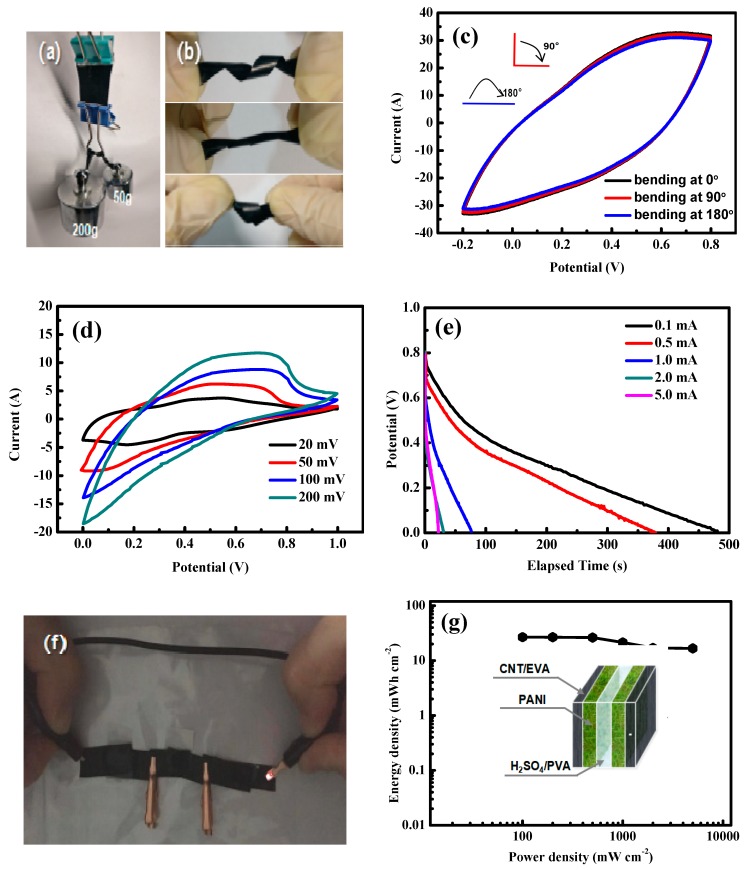
Digital photographs of the PANI/CNT/EVA electrode: (**a**) mass loading, (**b**) twisted into a spiral shape or tied into a knot, stretched, and released. (**c**) CV curves of the PANI/CNT/EVA electrode under different bending angles. (**d**) CV and (**e**) discharge curves of solid-state symmetric supercapacitors at different scan rates and current densities. (**f**) Digital photographs of the assembled flexible solid-state symmetric supercapacitors (3 in series) lighting LEDs. (**g**) Ragone plots of the areal energy density and power density, and the structure of flexible solid-state symmetric supercapacitors.

The presence of a nanostructured PANI-CNT architecture in the PANI/CNT/EVA composite film has almost no effect on the flexibility of the electrode. To further understand the tensile strength of the PANI/CNT/EVA, the load-bearing capacity of the film was also measured through a mass loading experiment. A half piece of film was sandwiched between two book clamps, as shown in Figure 5a. The electrode was well maintained under a load of 250 g (far more than its own mass), which proves that the PANI/CNT/EVA was robust with a high load-bearing capacity. As a result, the nanostructured PANI/CNT/EVA can easily be made into various shapes (Figure 5b) such as spiral stripes and knots, and can be stretched repeatedly and return to its initial state without any cracks (the SEM show in Figure S5). The PANI/CNT/EVA electrodes maintains good capacitance retention under different bending angles, reflecting the superior flexibility of the electrode. The flexible device was also bent from 0° to 180° repeatedly several times (Figure 5c). There was no obvious capacitance loss after bending. The structure of flexible solid-state symmetric supercapacitors is shown in Figure 5g. A solid-state symmetric supercapacitor was directly assembled using the PANI/CNT/EVA composite electrodes. The constructed solid-state symmetric textile supercapacitor is highly flexible and can be bent and folded without destroying the structural integrity of the devices. It can light a light-emitting diode (LED) for more than 3 min without dimming from visual observation (as shown in Figure 5d), which indicates a good energy storage ability. Figure 5d shows the CV curves of the PANI-50 solid-state symmetric supercapacitors collected at different scan rates ranging from 20 to 200 mV·s^−1^ in a voltage window from 0 to 1.0 V. No obvious distortion in the shape is observed at a high scan rate of 200 mV·s^−1^, indicating the fast charge/discharge properties. From the discharge curves (Figure 5e) we can determine the single areal capacitance of 620.1 mF·cm^−2^ (189.2 mF·cm^−2^ for device) at a current density of 0.5 mA·cm^−2^, and 306.4 mF·cm^−2^ (122.4 mF·cm^−2^ for device) at a current of 2 mA·cm^−2^. This indicates a capacitance retention of 64.7%, implying a significantly high power output capability. The energy density and power density of the as-prepared PANI-50 solid-state symmetric supercapacitors were also calculated to evaluate their ability in practical applications (Figure 5e). PANI-50 displays the highest areal energy density of 26.7 mWh·cm^−2^ with an areal power density of 100 mW·cm^−2^. The supercapacitor retains an excellent energy density of 16.7 mWh·cm^−2^ when the power density reaches 2400 mW·cm^−2^. 

## 4. Conclusions

In summary, we reported a process for the design and fabrication of PANI/CNT/EVA polymer conductive film. The good solubility of EVA in THF, carbon nanotubes combining well with the EVA particles, and choosing the facile electrodeposition method, increased the binding force between the PANI and the flexible substrate. This can improve the conductivity of the electrode and limit the volume change upon the charge/discharge process. The as-prepared PANI/CNT/EVA exhibited excellent long-term stability, with 66.4% capacitance retention. In addition, the PANI coverage not only improved the rate performance of the electrode, but also enhanced the specific capacitance up to 620.1 mF·cm^−2^. The assembled solid-state supercapacitor shows both outstanding flexibility and high-energy areal density of 26.7 mWh·cm^−2^ at a power density of 100 mW·cm^−2^, without showing any decline in performance under bending conditions. The demonstrated composite supercapacitor has appealing potential in many fields, such as in wearable equipment. 

## Supplementary Materials

The following are available online at http://www.mdpi.com/2073-4360/11/1/178/s1, Figure S1: SEM image of CNT/EVA-cotton, Figure S2: SEM image of 331 carbon cloth, Figure S3: SEM image of the PANI-50 electrode after 3000 cycles at a current density of 2 mA·cm^−2^, Figure S4: GCD curves of first and last 10 cycles during the 3000 cycles test. (PANI-50 electrode), Figure S5: SEM image of PANI-50 after repeated stretching.
