# Supplementary material for: In Situ Growth of a High-Performance All-Solid-State Electrode for Flexible Supercapacitors Based on a PANI/CNT/EVA Composite
Source: Polymers (Basel). 2019 Jan 21;11(1):178. doi: 10.3390/polym11010178 (PMC6401819; doi:10.3390/polym11010178)
Supplement: Supplementary file 1 [file polymers-11-00178-s001.pdf]

Supplementary Materials

In-Situ Growth of High-Performance All-Solid-State Electrode for Flexible Supercapacitors Based on PANI/CNT/EVA Composite

Xipeng Guan, Debin Kong, Qin Huang, Lin Cao, Peng Zhang, Huaijun Lin, Zhidan Lin*, Hong Yuan*,

Jinan University, Guangzhou 510632, China;
guanxipeng@jnu.edu.cn(X.G.), 13580457530@163.com(D.K.); hq2502@126.com(Q.H.); linc19993@163.com(L.C.); tzhangpeng@jnu.edu.cn (P.Z.); hjlin@jnu.edu.cn(H.L.)

* Correspondence: linzd@jnu.edu.cn(Z.L.); tyuanhong@jnu.edu.cn(H.Y.); Tel.: +86-020-8522−0890

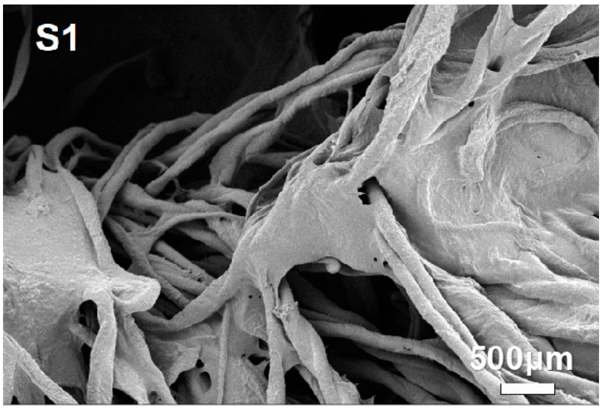

Figure 1. SEM image of CNT/EVA-cotton.

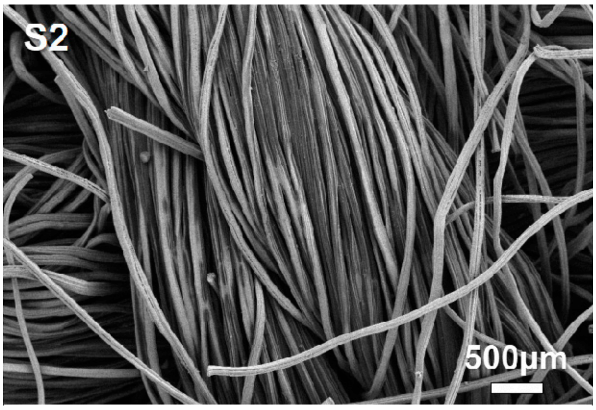

Figure S2. SEM image of 331 carbon cloth.

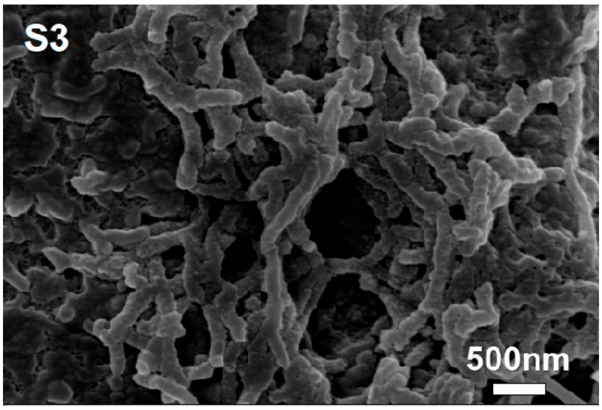

Figure S3. SEM image of the PANI-50 electrode after 3000 cycles at a current density of 2 $mA \cdot cm^{-2}$.

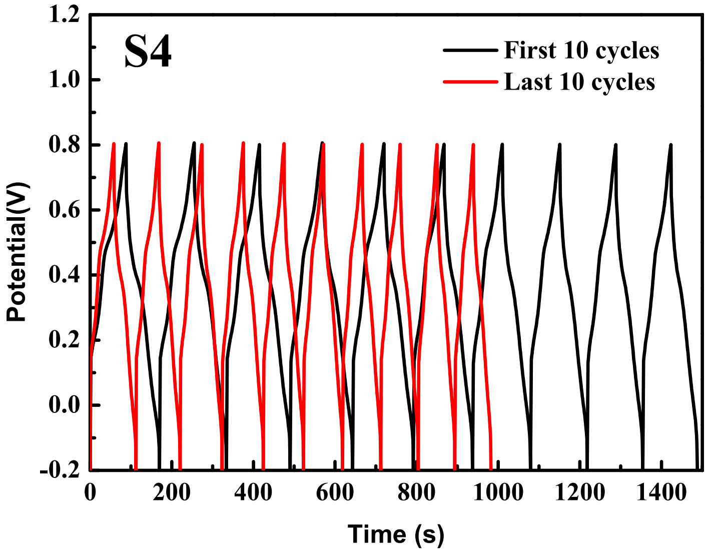

Figure S4. GCD curves of first and last 10 cycles during the 3000 cycles test.(PANI-50 electrode)

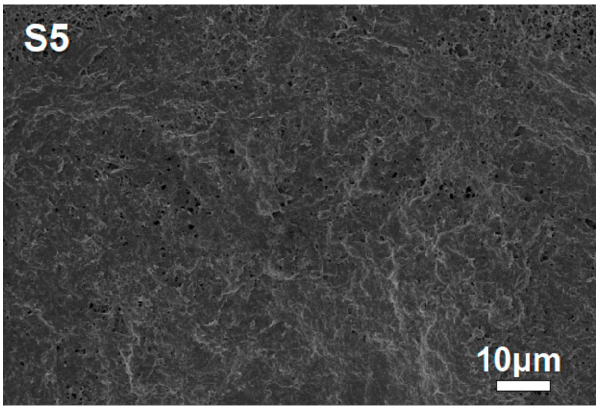

Figure S5. SEM image of PANI-50 after repeated stretching.
